# Chibby 1: a new component of β-catenin-signaling in chronic myeloid leukemia

**DOI:** 10.18632/oncotarget.21166

**Published:** 2017-09-22

**Authors:** Manuela Mancini, Simona Soverini, Gabriele Gugliotta, Maria Alessandra Santucci, Gianantonio Rosti, Michele Cavo, Giovanni Martinelli, Fausto Castagnetti

**Affiliations:** ^1^ Department of Experimental Diagnostic and Specialty Medicine, DIMES–Institute of Hematology “L. and A. Seràgnoli”, University of Bologna Medical School, Bologna, Italy

**Keywords:** chibby1, ß-catenin, ER stress, autophagy, BCR-ABL1

## Abstract

Chibby 1 (CBY1) is a small and evolutionarily conserved protein, which act as β-catenin antagonist. CBY1 is encoded by *C22orf2* (22q13.1) Its antagonistic function on β-catenin involves the direct interaction with:

The C-terminal activation domain of β-catenin, which hinders β-catenin binding with Tcf/Lef transcription factors hence repressing β-catenin transcriptional activation.

14-3-3 scaffolding proteins (σ or ξ), which drive CBY1 nuclear export into a stable tripartite complex with β-catenin.

The relative proximity of *C22orf2* gene encoding for CBY1 to the BCR breakpoint on chromosome 22q11, whose translocation and rearrangement with the c-ABL is the causative event of chronic myeloid leukemia (CML), suggested that gene haploinsufficiency may play a role in the disease pathogenesis and progression. We found CBY1 down-modulation associated with the *BCR-ABL1*, promoted by transcriptional mechanisms (promoter hyper-methylation) and post-transcriptional events, addressing the protein towards proteasome-dependent degradation through SUMOylation. CBY1 reduced expression in clonal progenitors and, more importantly, in leukemic stem cells (LSC), is contingent upon the tyrosine kinase (TK) activity of BCR-ABL1 fusion protein. Accordingly, its induction by Imatinib (IM) and second generation TK inhibitors contributes to β-catenin inactivation through multiple events encompassing the activation of endoplasmic reticulum (ER) stress-associated unfolded protein response (UPR) and autophagy, eventually leading to apoptotic death. These findings support the advantage of combined regimens including drugs targeting DNA epigenetics and/or proteasome to eradicate the BCR-ABL1+ hematopoiesis.

## INTRODUCTION

The “hierarchal clustering” model of tumors posits the key role of cancer stem cells (CSC), a pool of relatively quiescent cells otherwise named tumor initiating cells (TIC), in carcinogenesis. In this context, no tumor can be cured until the CSC pool has been eradicated. As the normal counterpart, CSC display self-renewal, express telomerase and activate anti-apoptotic and multi-drug resistance pathways [1]. The availability of *in vitro* culture techniques since the pioneering work of Till and McCulloch and the exhaustive phenotypic characterization of myeloid progenitor cells at different differentiation levels let define the ontogenesis of normal and transformed hematopoiesis [2, 3]. Finally, identification of the causative event of CML as the *BCR-ABL1* fusion protein TK let distinguish LSC from normal HSC, hence providing a host of information on signals involved in self-renewal, proliferation and life expectancy associated with leukemic transformation [4]. The most significant trait of CML LSC is *BCR-ABL1* independence, which makes them autonomous from the fusion protein TK for proliferation and survival and drives their resistance to TK inhibitors IM, Nilotinib and Dasatinib, hence providing a sanctuary for the disease relapse upon drug withdrawal and a putative source of drug-resistance [5]. Pro-survival signaling pathways of *BCR-ABL1*+ LSC have therefore attracted great interest in view of their use as pharmacological targets.

β-catenin is a central component of *BCR-ABL1*+ LSC self-renewal and microenvironment protection from TK inhibitors [6–9]. β-Catenin activation in CML is mostly contingent upon mechanisms hampering its degradation, including *BCR-ABL1*-mediated phosphorylation at specific tyrosine residues (Y86 and Y654), which prevents the recruitment by the adenomatous polyposis coli (APC)/Axin/glycogen synthase kinase 3 β (GSK3 β) destruction complex, the overexpression of growth arrest specific 2 (GAS2), which reduces calpain-dependent degradation, and GSK3β inactivation due to the prevalence of a GSK3β mis-spliced isoform unable to phosphorylate β-catenin and/or to GSK3β de-phosphorylation by the Fas-associated phosphatase 1 (Fap1) [10–13]. The prerequisite of β-catenin transcriptional activity is nuclear import and interaction with transcription factors of the TCF/LEF1 family and additional co-factors, such as B-cell lymphoma 9 (BCL9), cyclic AMP response element binding protein (CBP) and pygopus to regulate a series of target genes [14]. β-catenin nuclear transport is mediated by direct contact with the nuclear pore and regulated by phopshorylation and binding partners, including Forkhead box M1 (FOXM1), insulin receptor substrate (IRS-1), mucin 1 (MUC-1), BCL9, androgen receptor and LEF-1 [14]. Our review will be focused on the β-catenin antagonist CBY1.

CBY is a 14.5 kDa protein highly conserved throughout evolution. It directly interacts with the C-terminal activation domain of b catenin and competes with TCF/LEF factors for b catenin binding hence repressing its transcriptional activity [15]. Moreover, it forms a stable tripartite complex with 14-3-3z and b catenin hence promoting the b catenin nuclear exclusion and cytoplasmic compartmentalization [16, 17].

CBY1 participation in the constitutive activation of β-catenin in CML was suggested by our recent study showing that a significant reduction in CBY1 expression levels is associated with *BCR-ABL1* and correlates with nuclear b catenin increment [18]. The relative proximity of C22*orf*2 gene encoding for CBY1 to the BCR breakpoint on chromosome 22q11 as a consequence of deletions of distal BCR sequences occurring at the time of Philadelphia translocation suggested the gene haploinsufficiency eventually associated with disease worse prognosis [19]. Indeed, fluorescent *in situ* hybridization (FISH) did not let any evidence of C22*orf*2 loss associated with CML more advanced stage or worse prognosis. Conversely, we observed CBY1 down-modulation, driven by transcriptional and post-transcriptional mechanisms and evoked by the constitutive TK activity of *BCR-ABL1* fusion protein. CBY1 induction in *BCR-ABL1+* cell response to TK inhibitors triggers a series of events, including activation of the unfolded protein response (UPR) and autophagy eventually leading to selective leukemic cell death [20, 21]. Targeting signals involved in CBY1 down-modulation in CML may be, therefore, advanced as a complementary strategy to eradicate clonal hematopoiesis.

## CBY1 DOWN-MODULATION ASSOCIATED WITH *BCR-ABL1* TK CONTRIBUTES TO Β-CATENIN ACTIVATION IN LEUKEMIC HEMATOPOIESIS

CBY1 is a small conserved antagonist of β-catenin. It is encoded by the *C22orf2* gene at chromosome 22q13.1, downstream of BCR cluster region (22q11) involved in the t [9, 22] translocation [22]. CBY1 antagonistic function on β-catenin encompasses its direct interaction with the C-terminal activation domain of β-catenin (which hinders β-catenin binding with TCF/LEF transcription factors hence repressing β-catenin transcriptional activation) and 14-3-3 scaffolding proteins (s or z, which drives CBY1 nuclear export into a stable tripartite complex with β-catenin) [15–17]. Accordingly, CBY1 “loss of function” has been involved in the pathogenesis of some types of cancers, such as colon carcinomas and pediatric ependymomas [23, 24]. Due to the relative proximity of *C22orf2* [22q13.1] to the breakpoint cluster region on BCR (22q11) we first investigated whether *C22orf2* haplo-insufficiency, originated by deletion(s) downstream of BCR sequences as a result of the t(9, 22) translocation, was correlated with CML prognosis [22]. However, fluorescent *in situ* hybridization (FISH) established that the full length *C22orf2* gene follows BCR sequences in CML myeloid progenitors, and relocates to the derivative chromosome 9 (der(9q)) in patients with the typical translocation t [9, 22] [q34;q11] or to the second fusion gene in patients with variant translocations [18, 19]. Still, CBY1 expression is reduced in hematopoietic progenitors of CML patients at clinical diagnosis compared to healthy donors and further lowered in the LSC (CD34+) compartment, where β-catenin provides a key signal for proliferation and survival [8]. Restored expression of CBY1 in CML patients at the time of complete or major molecular response during treatment with TK inhibitors (when the whole or major part of hematopoiesis is the normal, *BCR-ABL1*- one) supports CBY1 down-modulation as a trait of leukemic clone. Lack of correlation between CBY1 expression, disease prognosis and response to TK inhibitors seems to exclude its involvement in the disease progression [18]. Further investigation let establish the dependency of CBY1 down-modulation from *BCR-ABL1* TK. The fusion protein inhibition in response to IM induced, in fact, CYB1 expression, which, in turn, abolished the leukemic clone growth and survival advantage through events proceeding from β-catenin nuclear export and degradation in the cytoplasm, activation of ER stress-associated pathway known as UPR, which leads to apoptotic death, and induction of an autophagic pathway, which addresses β-catenin to proteasome-independent degradation [20, 21, 25].

CBY1 down-modulation associated with *BCR-ABL1* TK is driven by multiple events, including transcriptional mechanisms, caused by the gene promoter hyper-methylation, and post-transcriptional modifications involved in the ubiquitin-mediated degradation by proteasome [15–17].

## CBY1 DOWN-MODULATION ASSOCIATED WITH BCR-ABL1 IS MEDIATED BY GENE PROMOTER HYPERMETHYLATION AND PROTEIN INSTABILITY

DNA methylation consists in the attachment of methyl groups (CH_3_) at the 5′ carbon position of the cytosine ring. It predominantly occurs at high density CpG regions named CpG islands, which cover the transcriptional initiation sites of approximately 70% of annotated gene promoters [26]. It is promoted by a family of enzymes, the DNA methyltransferases (DNMTs, encompassing the *de novo* methyltransferases DNMT3a and DNMT3b and the maintenance methyltransferase DNMT1), which catalyze CH_3_ group transfer to establish a permissive landscape for methyl-binding (MBD) proteins, such as MeCP2, MBD1, MBD2 and MBD4, involved in transcriptional repression [27]. Indeed, DNA hyper-methylation is a critical epigenetic mechanism for transcriptional silencing of genes involved in cancer development and progression, including those controlling DNA repair, cell cycle, cell adhesion, apoptosis and angiogenesis. Accordingly, it may be considered as a second hit in the Knudson's two-hit model of cellular transformation [28]. From a clinical perspective, such integrated view into cancer genomics might improve the therapeutic approach through DNA de-methylating agents. DNA hyper-methylation is a common event in CML and affects multiple genes [29]. We found that enhanced recruitment of DNMT1 at the *C22orf2* promoter is a component of CBY1 down-modulation in CML hematopoietic progenitors and LSC, hence suggesting the putative advantage of DNA-demethylating drugs, such as 5-Aza-CdR (also referred to as decitabine), in the disease therapy [30].

The reduction of protein stability is a further mechanism driving CBY1 down-modulation in CML. As CBY1 reduced transcription, the protein instability is mediated by *BCR-ABL1* TK activity through events affecting CBY1 binding with 14-3-3. Those events includes phosphorylation by AKT at a critical residue of CBY1 (serine 20) for 14-3-3 binding and the impaired phosphorylation at a 14-3-3 residue (serine 186) by c-Jun N-terminal kinase (JNK) [17, 20]. The enhanced degradation of CBY1 is driven by the ubiquitin proteasome system (UPS) through a complex and tightly controlled process encompassing the covalent attachment of K48-linked polyubiquitin chain to flag target proteins for degradation through the 26S proteasome [31]. In particular, a post-translational modification which utilizes small ubiquitin-like modifier (SUMO) groups to covalently attach target substrates and promote their ubiquitination and degradation has been involved in CBY1 increased degradation associated with BCR-ABL1 [17, 32]. These findings suggest that inhibitors of AKT/mTOR axis, 14-3-3 binding or proteasome have the potential to attenuate β-catenin signalling and may be, therefore, tested for clinical use. Notably, pilot studies support the synergistic effects of proteasome inhibitors and TK inhibitors on BCR-ABL1+ cells, including LSC [33, 34].

## CBY1 INDUCTION IN BCR-ABL1+ CELLS ACTIVATES UPR AND TRIGGERS THE EXECUTION PHASE OF APOPTOSIS

β-catenin nuclear exclusion and transcriptional inactivation is the major consequence of CBY1 enforced expression following *C22orf2* stable transfection and CBY1 induction in response to IM in *BCR-ABL1*+ cells [18]. In such a cell context, as in other cell types, CBY1-driven cytoplasmic re-location of β-catenin activates UPR, which, in turn, induced the BCL2-interacting mediator of cell death (BIM), hence contributing to the execution phase of apoptotic death [20].

UPR is a homeostatic signaling network that transduces information about the protein-folding status in the ER lumen to buffer fluctuations in the unfolded protein load and let the recovery of ER function. Under physiological conditions it acts as an adaptive mechanism to promote cell survival, while under high-level or chronic ER stress, it becomes overshadowed by alternative signals which commit cells to degeneration and culminate in apoptosis [35]. UPR occurs via three mechanisms: i) reduced translation of misfolded proteins, ii) enhanced translation of ER chaperones and iii) ER-associated degradation (ERAD) of misfolded proteins, which are transferred from the ER to the cytoplasm for subsequent ubiquitination and degradation by the 26S proteasome. UPR is triggered by the activation of three trans-membrane ER proteins: pancreatic ER kinase (PERK), inositol-requiring enzyme 1a (IRE1a) and activating transcription factor 6 (ATF6), whose oligomerization at the ER luminal domain activates downstream activities, to transduce life or death signals [36]. We proved that CBY1 enforced expression as well as its induction in response to IM activate PERK and IRE1a, which, in turn, trigger specific transcription factors to up-regulate their target genes. Activated PERK phosphorylates the eukaryotic translation initiator factor 2a (eIF2a) to slow down mRNA translation and protein synthesis [37]. Moreover, it allows selective translation of the activating translation factor 4 (ATF4) to induce transcription of the C/EBP-homologous protein (CHOP, otherwise termed GADD153), which inhibits expression of the anti-apoptotic BCL-2 to hasten cell death [38]. More importantly, CHOP induces transcription of BIM, a crucial tumor suppressor gene for CML response to IM [39, 40]. BIM participates in the death signal transmission from ER to mitochondria, hence contributing to the execution phase of apoptosis through the activation of ER resident caspase 12 [40]. Finally, CHOP induction inhibits β-catenin/TCF-dependent transcriptional activation and may, therefore, contribute to cyclin D1 reduction to promote BCR-ABL1+ cell growth arrest [41]. Still, the role of UPR in the survival of CML progenitors and LSC is elusive. Three recently published studies established the pro-survival effects of UPR on *BCR-ABL1*+ cells, hence raising the question of the fusion protein impact on individual UPR branches [42–44]. Further investigation is required, in particular, to elucidate the misfolded nature of *BCR-ABL1* protein, whether and how its expression and phosphorylation levels are involved in UPR activation, and the participation of *BCR-ABL1* downstream targets, such as JNK and AKT, in the induction of UPR sensors and effectors [36].

## CBY1-INDUCED AUTOPHAGY PARTICIPATES IN *BCR-ABL1*+ CELL COMMITMENT TO APOPTOTIC DEATH

Autophagy is a further consequence of CBY1 enforced expression and induction in response to IM in BCR-ABL1+ cells [21]. Autophagy is a self-catabolic process wherein bulk cytoplasmatic components such as aggregated/misfolded proteins and organelles are sequestered within double- or multi-membrane vesicles (autophagosomes) and then delivered to lysosomes for degradation. It may either serve as a cell death mechanism (otherwise named type II programmed cell death) or play a pro-survival role as part of an adaptive and detoxifying process in response to sub-lethal stress such as starvation, hypoxia, heat shock and microbial pathogens, and contingent upon the cell context and oncogenic status [45, 46]. In *BCR-ABL1*+ cells autophagy has been regarded as a complementary pathway to apoptotic cell death response to IM and other TK inhibitors proceeding from ER stress and ER Ca^2+^ store mobilization [47, 48]. More recently, autophagy has been implicated in normal and cancer stem cell self-renewal and survival in the low-oxygen “niche” of bone marrow microenvironment as well as in their quiescence through the m-TOR complex 1 (m-TORC1)-mitochondria-reactive oxygen species (ROS) axis. Moreover, it protects *BCR-ABL1*+ LSC from the lethal effects of TK inhibitors, hence contributing to the disease persistence. Indeed, cells expressing the *BCR-ABL1* rearranged gene of CML exhibit low basal levels of autophagy, but are highly dependent on autophagy for response to stress and leukemia induction in allograft models [49–52]. In a recently published study we showed that the autophagic phenotype originated in *BCR-ABL1*+ cells by the elevation of free Ca^2+^ release from ER stores in response to IM generates a 28 kDa cleaved calpain fragment which, in turn, promotes the cleavage of the ER-resident caspase 12 into a 42 kDa fragment corresponding to its activated isoform hence contributing to apoptosis commitment shown in a previously published study [21]. Notably, β-catenin is a calpain target [53]. Accordingly, its decrease following CBY1 induction in response to IM may be partly mediated by calpain activation and autolysosomal clearance upon autophagy induction [54]. It is worth to mention the calpain-mediated cleavage of β-catenin accumulated within the cytoplasmic compartment into a 75 kDa fragment still owning TCF-dependent transcriptional activity [55, 56]. Such β-catenin fragment may be regarded as the putative mediator of autophagy pro-survival effects in *BCR-ABL1*+ cells following IM treatment. Further investigation is required to identify signals directing autophagy impact on cell decision between life and death in a *BCR-ABL1*+ cell context.

## CONCLUSIONS

**Figure 1:**
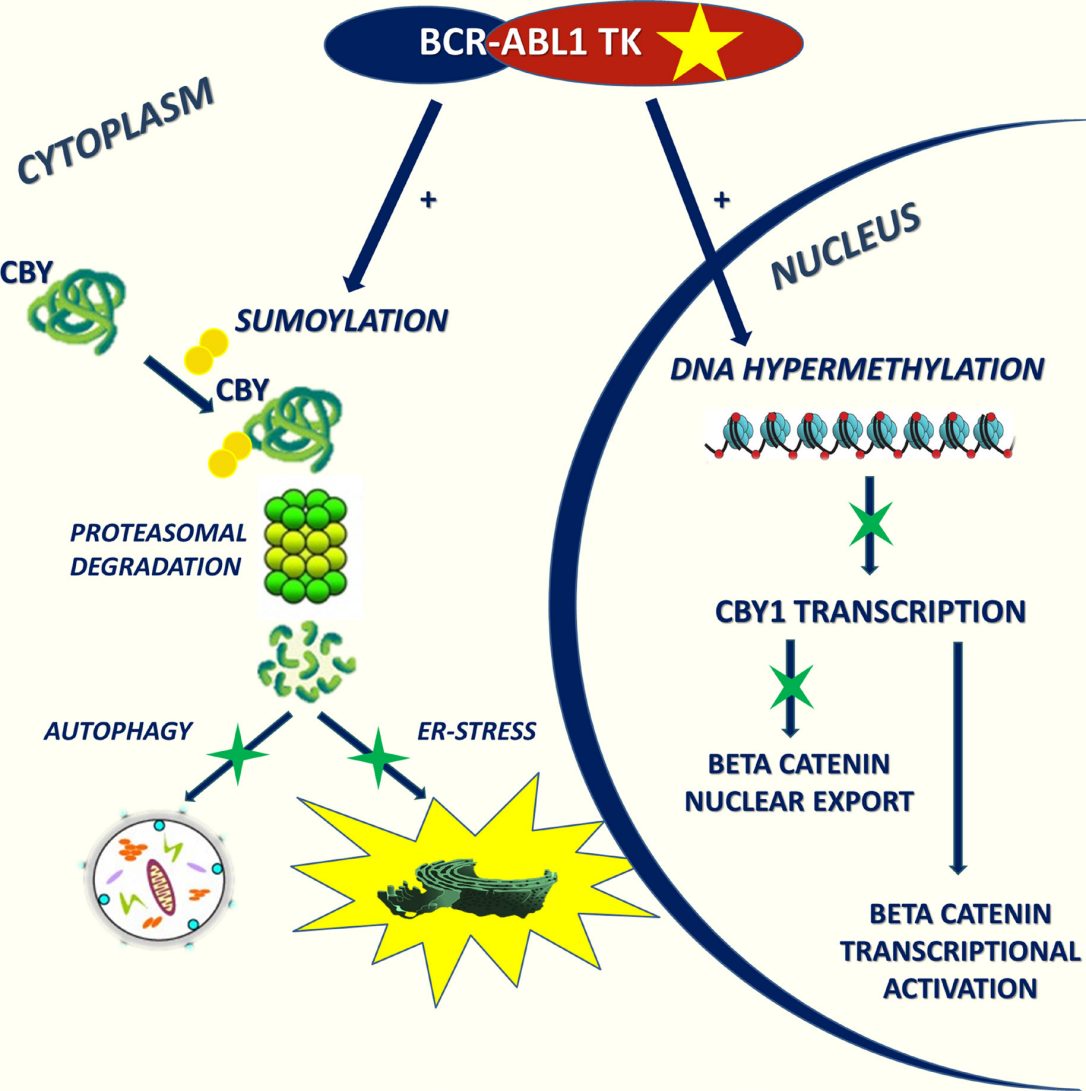
CBY1 down-modulation associated with BCR-ABL1 TK promotes β-catenin retention in the nucleus and its transcriptional activation It is driven by transcriptional and post-transcriptional events involving DNA hyper-methylation and protein enhanced degradation. Through its effects on β-catenin sub-cellular localization, CBY1 impacts UPR and autophagy in clonal hematopoietic progenitors.

Here we report of a new component of β-catenin network in CML (Figure 1). CBY1 down-modulation associated with *BCR-ABL1* TK promotes β-catenin retention within the nuclear compartment and transcriptional activation. It is driven by transcriptional and post-transcriptional events involving DNA hyper-methylation and protein enhanced degradation. Through its effects on β-catenin sub-cellular partitioning, CBY1 impacts UPR and autophagy in clonal hematopoietic progenitors and, more importantly, in LSC. The activation of UPR and autophagy may have a role in the balance between life and death of *BCR-ABL1*+ cells in response to TK inhibitors.
